# Parsimonious estimation of hourly surface ozone concentration across China during 2015–2020

**DOI:** 10.1038/s41597-024-03302-3

**Published:** 2024-05-14

**Authors:** Wenxiu Zhang, Di Liu, Hanqin Tian, Naiqin Pan, Ruqi Yang, Wenhan Tang, Jia Yang, Fei Lu, Buddhi Dayananda, Han Mei, Siyuan Wang, Hao Shi

**Affiliations:** 1 State Key Laboratory of Urban and Regional Ecology, Research Center for Eco-Environmental Sciences, Chinese Academy of Sciences, Beijing, 100085 China; 2 University of Chinese Academy of Sciences, Beijing, 100049 China; 3 Schiller Institute of Integrated Science and Society, Boston College, Chestnut Hill, MA 02467 USA; 4 College of Forestry, Wildlife and Environment, Auburn University, Auburn, AL 36849 USA; 5 Department of Forest and Wildlife Ecology, University of Wisconsin-Madison, Madison, WI 53706 USA; 6 Department of Atmospheric Sciences, University of Illinois Urbana-Champaign, Urbana, IL 61801 USA; 7 Natural Resource Ecology & Management, Oklahoma State University, Stillwater, OK 74078 USA; 8 School of Agriculture and Food Sciences, The University of Queensland, Brisbane, QLD 4072 Australia; 9 Division of Environment and Sustainability, The Hong Kong University of Science and Technology, Hong Kong, 999077 China

**Keywords:** Atmospheric science, Environmental sciences

## Abstract

Surface ozone is an important air pollutant detrimental to human health and vegetation productivity, particularly in China. However, high resolution surface ozone concentration data is still lacking, largely hindering accurate assessment of associated environmental impacts. Here, we collected hourly ground ozone observations (over 6 million records), remote sensing products, meteorological data, and social-economic information, and applied recurrent neural networks to map hourly surface ozone data (HrSOD) at a 0.1° × 0.1° resolution across China during 2015–2020. The coefficient of determination (R^2^) values in sample-based, site-based, and by-year cross-validations were 0.72, 0.65 and 0.71, respectively, with the root mean square error (RMSE) values being 11.71 ppb (mean = 30.89 ppb), 12.81 ppb (mean = 30.96 ppb) and 11.14 ppb (mean = 31.26 ppb). Moreover, it exhibits high spatiotemporal consistency with ground-level observations at different time scales (diurnal, seasonal, annual), and at various spatial levels (individual sites and regional scales). Meanwhile, the HrSOD provides critical information for fine-resolution assessment of surface ozone impacts on environmental and human benefits.

## Background & Summary

Ozone (O_3_) is an important constituent of the atmosphere and is ubiquitously present in both the troposphere and the stratosphere. Stratospheric ozone protects life on Earth by absorbing harmful solar ultraviolet rays^1–3^. Tropospheric ozone is a major gaseous pollutant produced in a series of complex reactions between volatile organic compounds (VOCs) and nitrogen oxides (NOx) in the presence of sunlight^4^. Exposure to high-concentration surface ozone can cause severe impacts on human health, inducing high morbidity in respiratory, cardiopulmonary, and cardiovascular diseases^5–7^. Moreover, surface ozone of high concentrations could damage the leaf cell structure of plants and thus decrease natural vegetation productivity, crop yield and quality^8–11^.

In the past decades, the number of ozone pollution events has increased significantly, particularly in highly populated and developed regions^12–15^. Real-time surface ozone monitoring networks have been established on a regional basis around the world. But their coverage is still insufficient in both space and time, due to uneven distribution of monitoring sites and lack of mid- to long-term continuous records in the majority of the world^10,16^. In contrast, satellite remote sensing can monitor the spatial and temporal variability of ozone at regional to global scales. For instance, the Ozone Monitoring Instrument (OMI) on the Aura satellite, launched in 2004, provides global daily total column ozone retrievals. Nonetheless, satellite-based estimates of surface ozone concentrations are not available at high spatial and temporal resolutions^17,18^. Hence, various models have been developed to extrapolate site observations, refine satellite retrievals, or fuse them to generate long-term, high-quality surface ozone datasets^19,20^.

These models, according to their underlying principles, can be generally grouped into chemical transport models (CTMs), geostatistical models, and machine learning models. CTMs are physics-based, accounting for atmospheric chemical reactions, emission inventories, meteorological conditions and transport of atmospheric pollutants, but usually are prone to high uncertainties in emission inventories and model assumptions^21–23^. Geostatistical models, such as Kriging interpolation^24^, land-use regression (LUR), Bayesian maximum entropy^25^ (BME), and geographically weighted regression^26^ (GWR), estimate surface ozone by fitting its relationships with the influential factors. However, collinearity (the non-independence of predictor variables) in these geostatistical models usually makes them difficult to estimate accurately^19,27^. Machine learning models, such as neural network, random forest (RF) and extreme gradient boosting (XGBoost), are widely used due to their strong data-mining capabilities. Among them, deep learning algorithms utilize more precise hidden layer structures for data-driven prediction, resulting in higher prediction accuracy than traditional regression and neural network models^28^, and have been developing rapidly and show great potential for predicting atmospheric pollutions including surface ozone concentrations. For instance, Eslami *et al*.^29^ utilized a deep convolutional neural network (CNN) to predict hourly ozone concentrations in Seoul, South Korea in 2017. Cheng *et al*.^30^ used a hybrid deep learning model to explore the complex nonlinear relationships between meteorological factors and ozone concentrations and applied it to hourly and daily forecasts of ozone concentrations in China.

In recent years, surface ozone pollution in China has become increasingly serious, with frequent large-scale high ozone pollution events^31–33^. Since 2013, China has established a national ozone observation network^10^, utilizing which several gridded surface ozone products were generated^34,35^. Liu *et al*.^19^ utilized the XGBoost algorithm in combination with monitoring station data, concurrent ozone retrievals, aerosol reanalysis, meteorological parameters, and land use data to predict maximum daily average 8-hour ozone (MDA8) concentration across China from 2015 to 2020. At the daily level, the coefficient of determination (R^2^) values for cross validation (CV) were 0.61–0.78. Wang *et al*.^33^ used a space-time extremely randomized trees (STET) model, with solar radiation intensity and air temperature as the main predicting factors, combined with ground observation data, meteorological data, and emission inventory data, to simulate MDA8 data across China from 2013 to 2020, with R^2^ of 0.87 and the root mean square error (RMSE) of 17.10 µg m^−3^. However, some input variables, particularly those related to ozone precursor emission inventories, were found to contribute less significantly than originally anticipated^20^. Moreover, the predictions were mostly focused on daily ozone concentrations, such as MDA8. Although there have been some exceptional datasets of hourly surface ozone concentrations^36^, long-term gridded hourly products of high accuracy are still lacking in China. Such a data gap impedes accurate assessment of environmental and human health impacts of surface ozone. For example, in estimating ozone damage to crops, hourly ozone data is usually required for stomatal ozone flux models^37^ or generating ozone exposure index^38,39^. Moreover, hourly ozone data is advantageous over that at coarser temporal resolution in determining ozone exposure of humans^40^.

To address the issue, here we developed a deep learning model based on the Long Short-Term Memory (LSTM) recurrent neural networks to generate hourly surface ozone data (HrSOD) at a spatial resolution of 0.1° × 0.1° from 2015 to 2020 over China. The model utilized a parsimonious set of predictor variables (excluding co-linear variables and ozone precursor emission inventories), including meteorological factors, remote sensing data, socio-economic and land use data, and more than six million ground station monitoring records as references.

## Methods

### Data

#### Surface ozone observation data

**Fig. 1:**
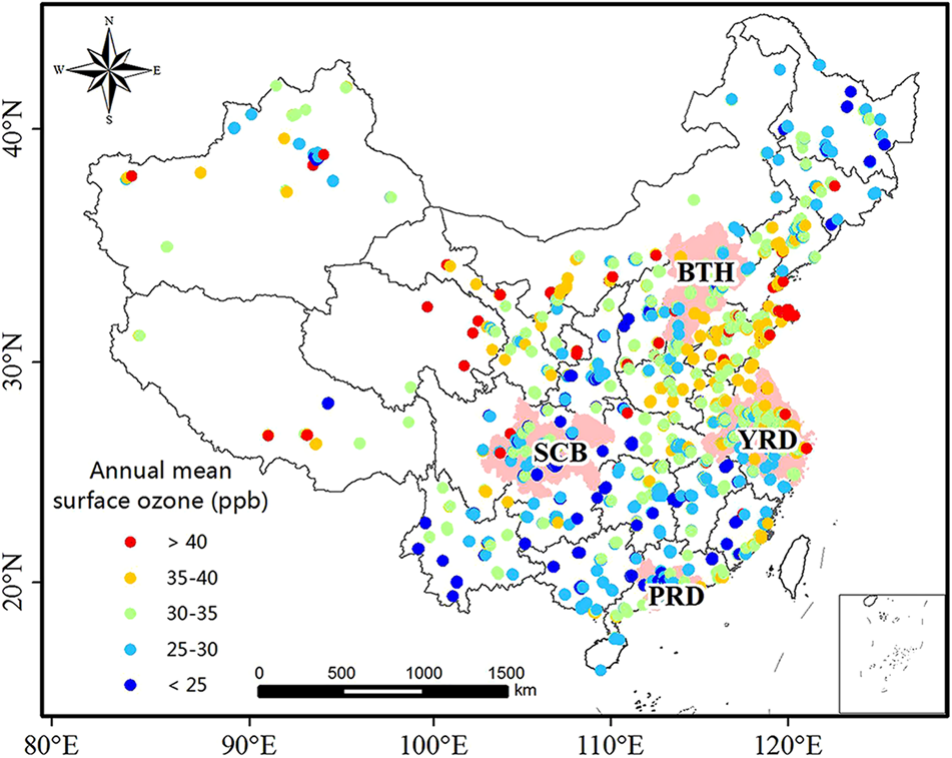
Spatial distribution of surface ozone observation sites in China. The color indicates the mean annual surface ozone concentration at each site during 2015–2020. The pink shaded regions indicate four megacity clusters of China, namely the Beijing-Tianjin-Hebei (BTH) region, the Pearl River Delta (PRD), the Sichuan Basin (SCB), and the Yangtze River Delta (YRD).

Over six million records of hourly surface ozone concentration measurements during June 2014 to February 2021 were obtained from the real-time air quality monitoring platform of the China National Environmental Monitoring Centre (CNEMC; https://air.cnemc.cn:18007/) and the archived data was uploaded to the Zenodo repository^41^ (10.5281/zenodo.10911197). The monitoring network was expanded to more than 1500 monitoring sites from 2013 to 2020, covering 31 provinces and 368 cities across mainland China. However, these monitoring sites are mainly located in the eastern region of China, with a much lower site distribution density in the northwest and the Qinghai-Tibet Plateau (Fig. 1).

Hourly ozone concentrations are measured at all monitoring sites by continuous monitoring instruments, and the unit of ozone reported by CNEMC is µg m^−3^ (standard atmospheric conditions at a temperature of 273.0 K and a pressure of 1013.25 hPa; 1 µg m^−3^ = 0.467 ppb). According to the Ambient Air Quality Standard^42^ and the Technical Specification for Ambient Air Quality Assessment^43^ set by the Ministry of Ecology and Environment of China (MEE) for ozone concentration data norms and standards, the ozone data was screened by removing outliers and null values. The multi-year mean hourly ozone concentrations ranged from 14–48 ppb during 2015–2020 in China, with areas of high ozone concentrations mainly in eastern China, especially in four densely populated megacity clusters of China, including the Beijing-Tianjin-Hebei (BTH) region, the Pearl River Delta (PRD), the Sichuan Basin (SCB) and the Yangtze River Delta (YRD).

#### Predictor variables

The predictor variables include satellite retrieved ozone products, meteorological factors, land use, population, and gross domestic product (see Supplementary Table S1).

- **Remote sensing data**
  The OMI carried by the Earth Observing System (EOS) Aura satellite was launched by the United States in 2004. Its primary mission is to monitor trace gases in the atmosphere, such as ozone, sulfur dioxide and nitrogen dioxide, while also collecting information on aerosols, clouds, ozone profiles, etc. The OMI sensor operates in a wavelength range of 270 to 500 nm with a spectral resolution of 0.5 nm. It has a swath width of 2600 km and provides a spatial resolution of 13 km × 24 km. OMI can complete a global scan in just one day, measuring column concentrations and profiles of O_3_, NO_2_, SO_2_, as well as data on aerosols, clouds, surface ultraviolet radiation, and various other parameters^18,44^. Previous studies have shown that the OMI ozone column concentrations and profile data exhibit a reasonable consistency with lower- to mid-troposphere ozone across the world^17,45^. Similarly, the OMI ozone data for different cities in China also manifests a high consistency with ground measurements^46,47^, facilitating a wide range of applications in atmospheric ozone research^48,49^.
  We collected remote sensing data including OMI Level 3 global daily total ozone grid product^50^ (OMTO_3_G; https://disc.gsfc.nasa.gov) and ozone profile products (PROFOZ; v0.9.3, level 2), which is derived using backscattered radiation within the sensitive ultraviolet spectral range for various atmospheric constituents^51^. The OMI provides daily ozone column concentration (0.25° × 0.25°) data, and the ozone profile product contains 18 vertical layers^52^, of which the first layer (air pressure of 1000 hPa) was selected to represent surface ozone in this study. We also calculated the average percentage of days with valid OMI data for each grid cell from 2015 to 2020 (Supplementary Figure S1). Most grid cells had a relative high percentage of qualified OMI retrievals, ranging from 64% to 83%. Specifically, the central and eastern regions had an average percentage between 70% and 75%, while the northeastern region had a lower percentage. The percentage in the southern region showed a larger spatial variability.
- **Climate data**
  Meteorological conditions are key driving factors in the formation process of surface ozone at short time-scales^53–55^. A total of seven climatic variables (solar radiation, air temperature, relative humidity, surface pressure, horizontal wind velocity, vertical wind velocity, and precipitation) were obtained from the ERA5-Land reanalysis data (Supplementary Table S1). Air temperature and solar radiation, which contribute to photochemical reactions, have strong positive correlations with ozone concentration^56–58^, whereas there is a significant negative correlation between ozone concentrations and atmospheric pressure. When the near surface is controlled by low pressure, pollutants from surrounding areas converge towards the center, driven by high-pressure air masses, resulting in a sharp increase in ozone concentrations in the center of the low pressure^59^. The relative humidity is negatively correlated with ozone concentrations because high relative humidity generally corresponds to precipitation, fog, and other weathers that do not have strong UV radiation, which is not conducive to the occurrence of photochemical reactions and the further development of ozone pollution^60^. The impact of wind speed on surface ozone concentration is complex. High wind speeds can lead to the dilution of local ozone concentrations, resulting in a negative correlation with the concentrations. However, high wind speeds can also enhance the transport of pollutants downwind, resulting in a positive correlation with downwind ozone concentrations^61^. As for precipitation, it facilitates the removal of pollutants such as ozone^58,62^.
  The ERA5-Land reanalysis dataset has a spatial resolution of 0.1° × 0.1° (about 9 km) and an hourly time-step, produced by the European Centre for Medium-Range Weather Forecasts (ECMWF; https://www.ecmwf.int/en/forecasts). The ERA5 reanalysis data combines land surface model simulations with ground and satellite observations^63,64^, and has been widely used across the world^65^. It has also been validated in China, showing good performance in predicting air temperature^66^, solar radiation^67^, and precipitation^68^.
- **Auxiliary data**
  Socio-economic data reflects human living and production activities, which is the major source of ozone precursors^4^ (VOCs and NOx). Existing emission inventories have significant uncertainties and low temporal resolutions, largely restricting their use in predicting hourly surface ozone concentrations^69^. we used socio-economic data and land use data as an input. We obtained population distribution data and Gross Domestic Product (GDP) data with a 1 km spatial resolution from the Resource and Environmental Science and Data Center, Chinese Academy of Sciences^70^ (https://www.resdc.cn/DOI). The data has a time interval of five years and is available for two years (2015 and 2019) during the study period. The nationwide land use data was derived from the Moderate Resolution Imaging Spectroradiometer^71^ (MODIS; https://lpdaac.usgs.gov/products/mcd12c1v006/) product at a resolution of 0.05°. Additionally, we also included latitude and longitude as predictor variables.
- **Data processing**

**Fig. 2:**
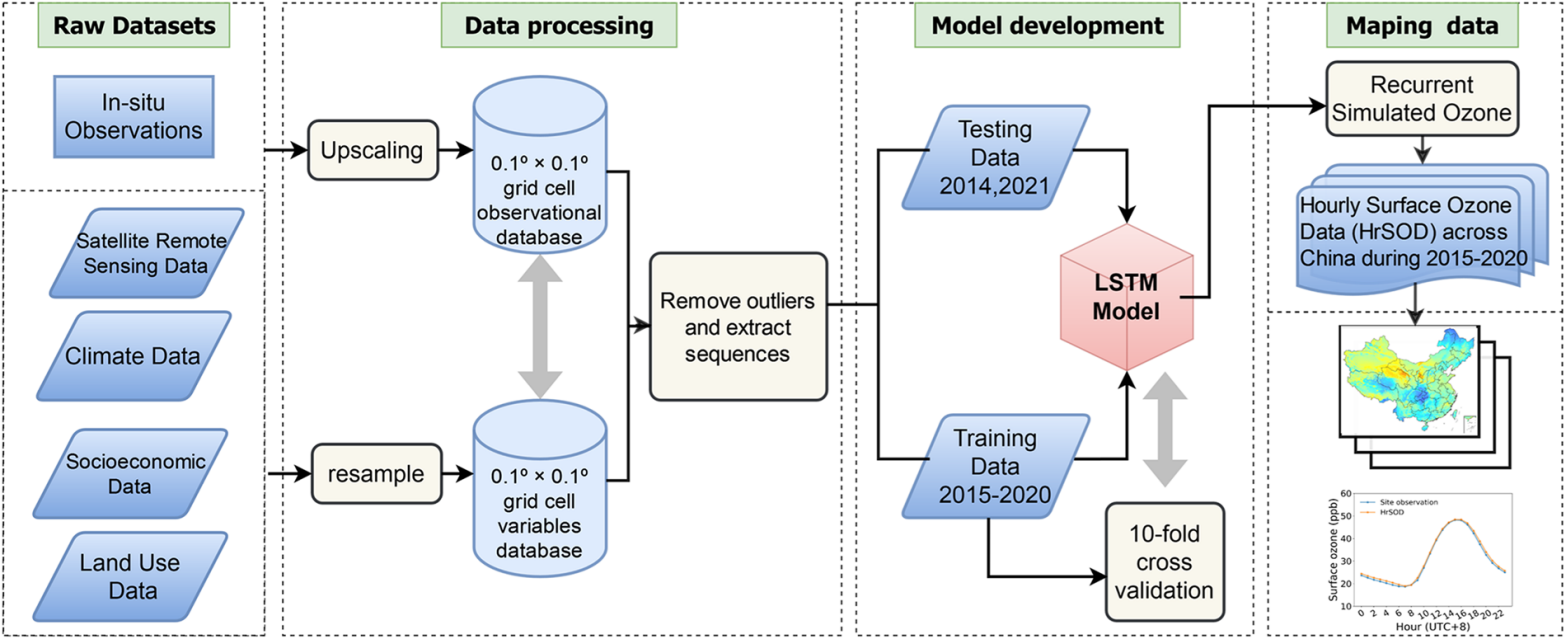
Flowchart for generating hourly surface ozone data (HrSOD) across China.

We constructed a 0.1° × 0.1°grid over China and averaged all the concurrent surface ozone measurements of monitoring sites within each grid cell to obtain grid-level surface ozone concentrations. Finally, we obtained a total of 643 grid cells with surface ozone observations across China (Fig. 2). The Thin Plate Spline (TPS) method was used to fill the missing values in OMI total column ozone data (Supplementary Figure S2). TPS has been proven to be effective in interpolating meteorological data^72^ and handling missing values in OMI remote sensing data, including total column ozone^73^ and aerosol optical depth^74^. Correspondingly, all predictor variables (including satellite retrievals, climate, land use, population distribution, and GDP data) were aggregated or resampled to the targeted grid resolution of 0.1° × 0.1° using the nearest neighbor interpolation or the bilinear interpolation approach. To avoid high collinearity among predictor variables, we conducted variance inflation factor (VIF) tests to all the predictor variables and only those with a VIF value less than 8.0 were retained^30^ (Supplementary Figure S3).

### Model development

#### The long short-term memory network model

The long short-term memory network is a special type of recurrent neural networks (RNN) that differs from traditional ones. The traditional artificial neural network (ANN) is fully connected between layers and has no connection within a specific layer, whereas the hidden layers of RNN are connected^75^. The output of RNN is not only affected by the current input features but also influenced by the output of the previous moment, and thus RNN generally has a better performance in estimating time-series and has been widely used to proceed sequence data^76^.

The LSTM can further overcome the limitations of conventional RNNs that they could be trapped by a vanishing gradient or exploding gradient during training^75^. It excels through integrating input gates, forgetting gates, and output gates into the cell structure. The input gates control whether a cell value can be added to a memory cell, the forgetting gates determine the weight of the value, and the output gates determine which information eventually is output from the cell. The LSTM has a long-term memory capability, which is ideal for predicting long time-series of historical ozone concentrations.

Specifically, based on LSTM, we built a five-layer neural network model for surface ozone concentration prediction (Supplementary Figure S4). It consists of an input layer, two LSTM layers, one Dense layer (also called fully connected layers), and an output layer. The data specification for the model’s input layer is in a 3-dimensional format (n_samples, n_time_steps, n_features), where n_samples represents the batch size for training, n_time_steps is the time window of 24 hours (to determine the optimal time window for training, we conducted several experiments with eight different lookback windows the detailed experiment results are shown in the Supplementary Table S2), representing the first 24 hours’ ozone sequence to predict the ozone at the 24th hour, and n_features is the number of 12 variables in the training set.

To determine the optimal hyperparameters (including epoch, batch size, number of neurons and optimizer), we first conducted a sensitivity analysis to identify the importance of each hyperparameter. Specifically, each hyperparameter was assigned a prior range, and the whole dataset was partitioned into the training data and the validation data using a ratio of 9:1. We adopted a one-at-a-time (OAT) strategy, i.e., changing one parameter at a fixed interval while keeping others unchanged, to avoid consuming too many high-performance computer resources. The results showed that changes in hyperparameters had minor effects on model performance (the R^2^ and RMSE values were nearly stable, being around 0.7 and 10.00 ppb, respectively). Thus, the mean value of the specific range for each of the hyperparameters was used. Supplementary Figure S5 shows the convergence of the loss function using the final hyperparameters. The number of neurons in each hidden layer is 50, and we used mean absolute error (MAE) as the loss function and the Adaptive moment estimation (Adam) as the optimization algorithm. The model was trained for 50 epochs with a batch size of 3000. The CNEMC ground measurements were used as the target for the model training and validation (Table 1).

**Table 1:** Detailed configuration of the neural network.

Configuration	Value
Training algorithm	Long Short-Term Memory (LSTM)
Number of hidden layers	3
Number of neurons in a hidden layer	50
Number of input variables	12
Number of output variables	1
Training data percentage	90%
Validation data percentage	10%
Data normalization	Minmax
Loss function	Mean absolute error (MAE)
Optimization algorithm	Adaptive moment estimation (Adam)

#### Model training, validation and test

All selected predictor variables that passed the VIF tests were used as inputs to train the LSTM model. The importance of different variables in the model was determined using the permutation importance method^77^, which measures the degree of decline in the model’s performance score after the random rearrangement of different features, and also represents the importance of each variable in estimating the concentration of ozone in the model. The feature importance scores of all selected variables in the pre-trained model are shown in Supplementary Figure S6. Air temperature, surface pressure and relative humidity were the top three factors affecting the spatiotemporal variability of surface ozone concentrations in China. In addition, longitude, day of year (DOY), latitude, downwelling surface radiation, wind speed, land use data, socio-economic data, and OMI’s SFO_3_ product also have significant impacts on ozone estimation. Finally, total column concentration ozone data and precipitation have relatively weaker influence in the model.

**Fig. 3:**
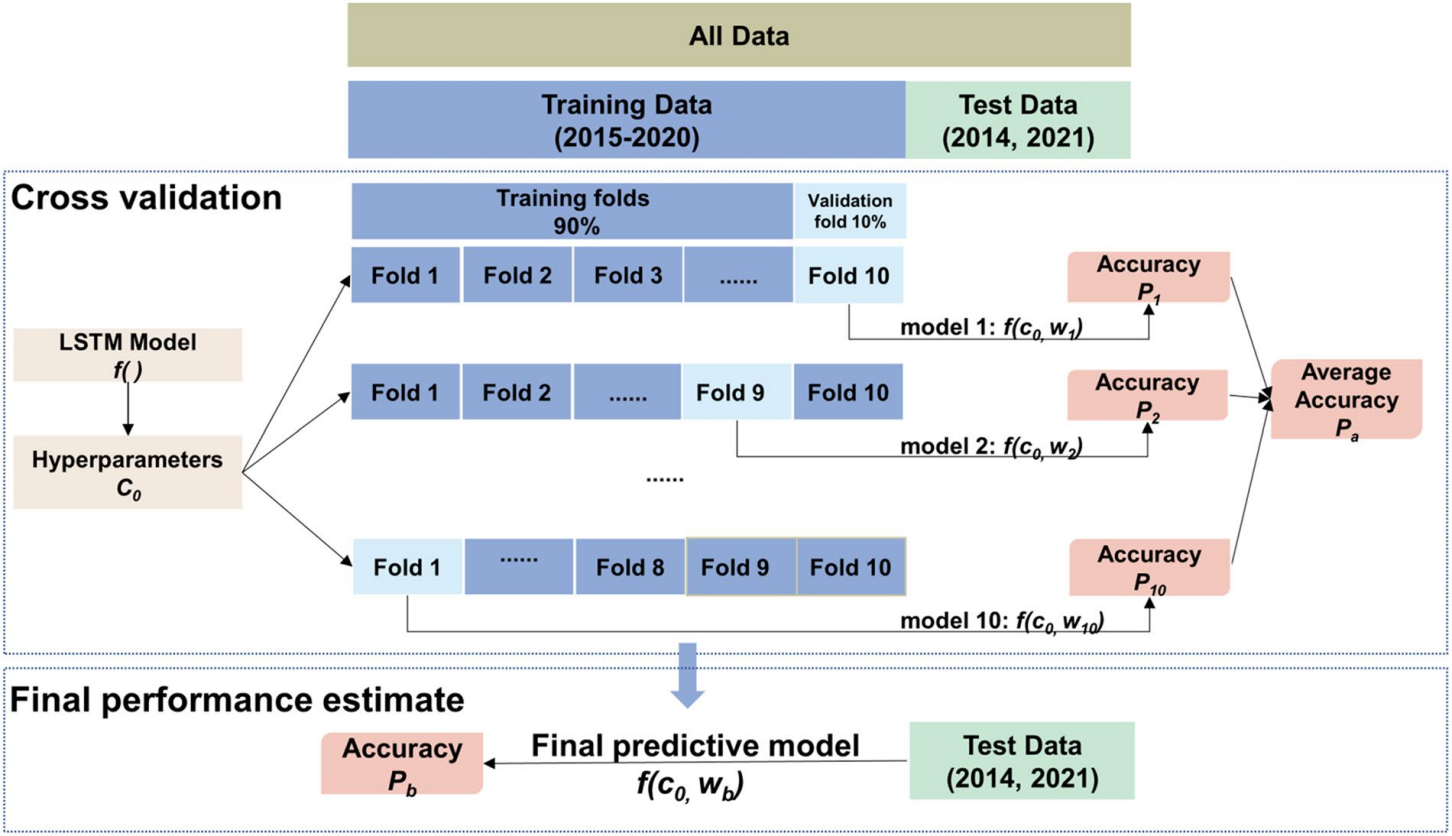
Detailed process of model cross-validation and testing.

We divided the original data into a training set (more than 600 grid cells during 2015–2020) and a testing set (for the periods of June to December in 2014 and January to February in 2021). To determine the best model and its corresponding hyperparameters, the 10-fold CV approach was utilized to evaluate the performance of the LSTM model on the training set (from 2015 to 2020), with three sampling strategies, namely sample-based CV, site-based CV and by-year CV, corresponding to the model’s performances on capturing overall, spatial, and temporal patterns, respectively. We performed a by-year CV by dividing the dataset into six folds, each representing one year from 2015 to 2020. In each iteration, five folds were used as the training set and the remaining fold was used as the validation set. The training and validation processes were repeated six times. In the other two sampling strategies, 90% of the total surface ozone observations were randomly sampled for training, and the rest 10% was used for validation, the process of which was repeated 10 times. In addition, ozone monitoring station data obtained in 2014 and 2021 was used as a test data set to evaluate the generic capability of the optimal LSTM model. The specific process is shown in Fig. 3.

The R^2^, RMSE, MAE, linear regression slope, and intercept were calculated to evaluate the performance of the model.

1 $${R}^{2}=1-\frac{{\sum }_{i=1}^{n}{\left({p}_{i}-{o}_{i}\right)}^{2}}{{\sum }_{i=1}^{n}{\left({p}_{i}-\bar{p}\right)}^{2}}$$

2 $$RMSE=\sqrt{\frac{1}{n}{\sum }_{i=1}^{n}{\left({p}_{i}-{o}_{i}\right)}^{2}}$$

3 $$MAE=\frac{1}{n}{\sum }_{i=1}^{n}\left|{p}_{i}-{o}_{i}\right|$$

Where the subscript *i* represents the pairing of *n* observed ozone concentrations *p*_*i*_ and their corresponding predictions *o*_*i*_, and $\bar{p}$ represents the arithmetic mean of the observed ozone concentrations.

## Data Records

The HrSOD dataset^78^ is available on the Zenodo repository at 10.5281/zenodo.7415326. The gridded ozone concentration data are provided in NetCDF format at 0.1° spatial resolution and hourly temporal resolution during 2015–2020 in ppb. The file size is 40 GB. The hourly data is a NetCDF file and the file is named “YYYYMMDD.nc”, where “YYYY”, “MM” and “DD” refer to the year, month, and data of the file. We have uploaded all the ozone site measurements data^41^ to the product repository. And this data can be accessed via the link: 10.5281/zenodo.10911197.

This study’s external data include OMI satellite remote sensing data for total column ozone^50^ and surface ozone concentrations^51^, available at 10.5067/Aura/OMI/DATA2025 and 10.5067/Aura/OMI/DATA2026 respectively. Climate data^65^ were sourced from ERA5-land at 10.24381/cds.e2161bac. Socio-economic information, such as population distribution and GDP data^70^, is accessible through http://www.resdc.cn/DOI. Nationwide land use data^71^ was derived from MODIS, available at https://lpdaac.usgs.gov/products/mcd12c1v006/.

## Technical Validation

### Model evaluation

**Fig. 4:**
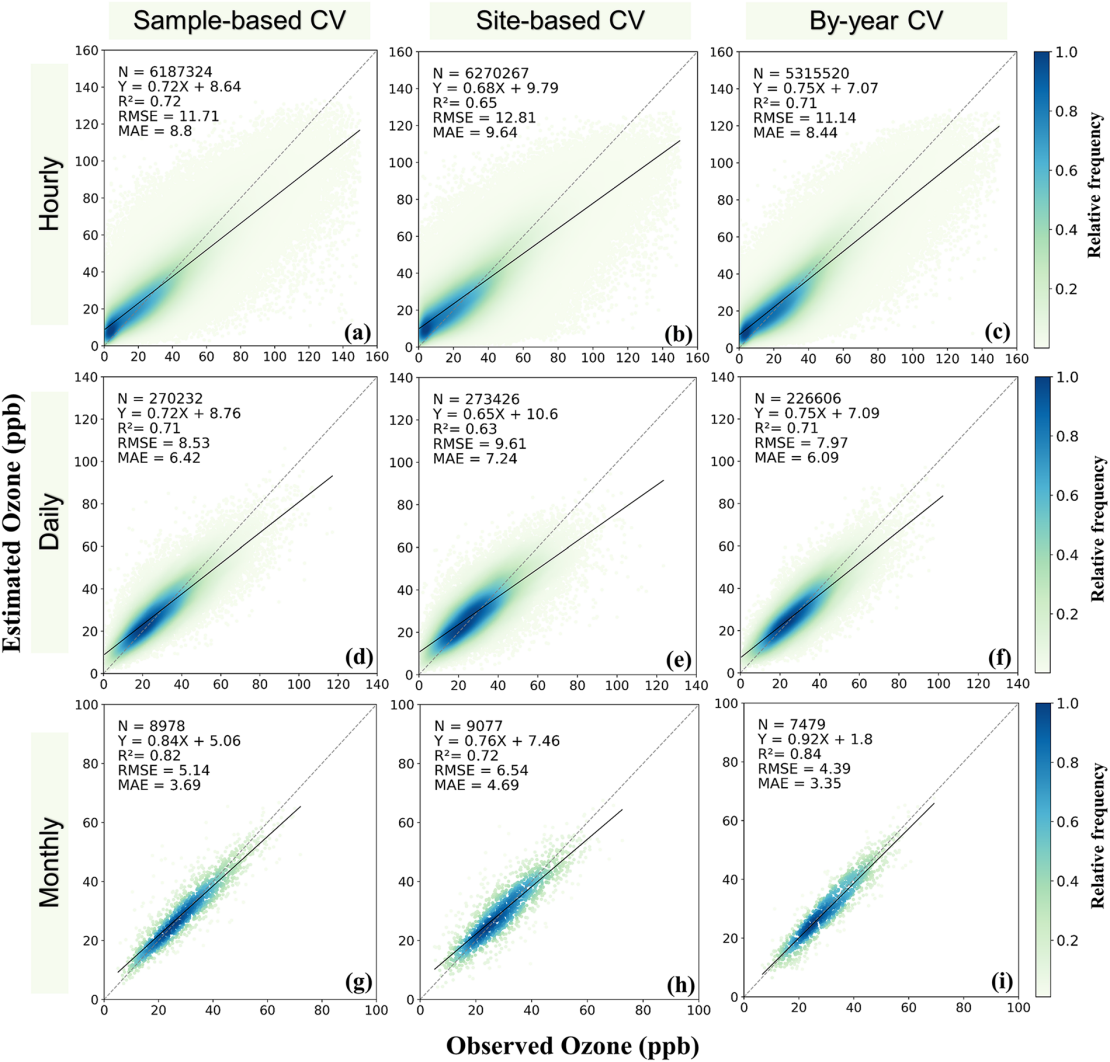
Comparisons between model estimated surface ozone concentrations and observations across China. The panels show sample-based cross validations at hourly, daily and monthly time-steps (**a,d,g**), site-based cross validations at hourly, daily and monthly time-steps (**b,e,h**), and by-year cross validations at hourly, daily and monthly time-steps (**c,f,i**). The dashed and black lines represent the 1:1 lines and the linear regression lines, respectively.

At the hourly time-scale, the LSTM model obtained R^2^ values of 0.72, 0.65 and 0.71 using three CV sampling methods (sample-based, site-based and by-year), respectively. The corresponding RMSE values were 11.71 ppb, 12.81 ppb, 11.14 ppb, and MAE values were 8.80 ppb, 9.64 ppb and 8.44 ppb (Fig. 4a–c). At the daily time-step, the model’s performance improved with R^2^ values of 0.71, 0.63, and 0.71 (sample-based, site-based, and by-year), RMSE values of 8.53 ppb, 9.61 ppb, and 7.97 ppb, and MAE values of 6.42 ppb, 7.24 ppb, and 6.09 ppb (Fig. 4d–f). The predictive ability of the model further improved at the monthly time-step, with higher R^2^ values of 0.82, 0.72, and 0.84 (sample-based, site-based, and by-year), smaller RMSE values of 5.14 ppb, 6.54 ppb, and 4.39 ppb and MAE values of 3.69 ppb, 4.69 ppb, and 3.35 ppb (sample-based, site-based, and by-year) (Fig. 4g–i).

Among the three CV sampling strategies, the site-based CV (Fig. 4b,e,h) had slightly lower R^2^ values than the sample-based CV (Fig. 4a,d,g) and by-year CV (Fig. 4c,f,i) R^2^ values, while the RMSE and MAE values were slightly higher than the sample-based CV RMSE and MAE values and by-year CV RMSE and MAE values. It is worth noting that the model tended to underestimate surface ozone when it was at high concentrations, but this bias was largely reduced at the monthly time-step (Fig. 4g–i). In addition, we compared the performance of LSTM with two other commonly used machine learning methods (RF and XGboost) using the same input data at an hourly time-step. The results show that the LSTM model performed better, particularly in terms of R^2^, RMSE, and slope values (Supplementary Figure S7).

**Fig. 5:**
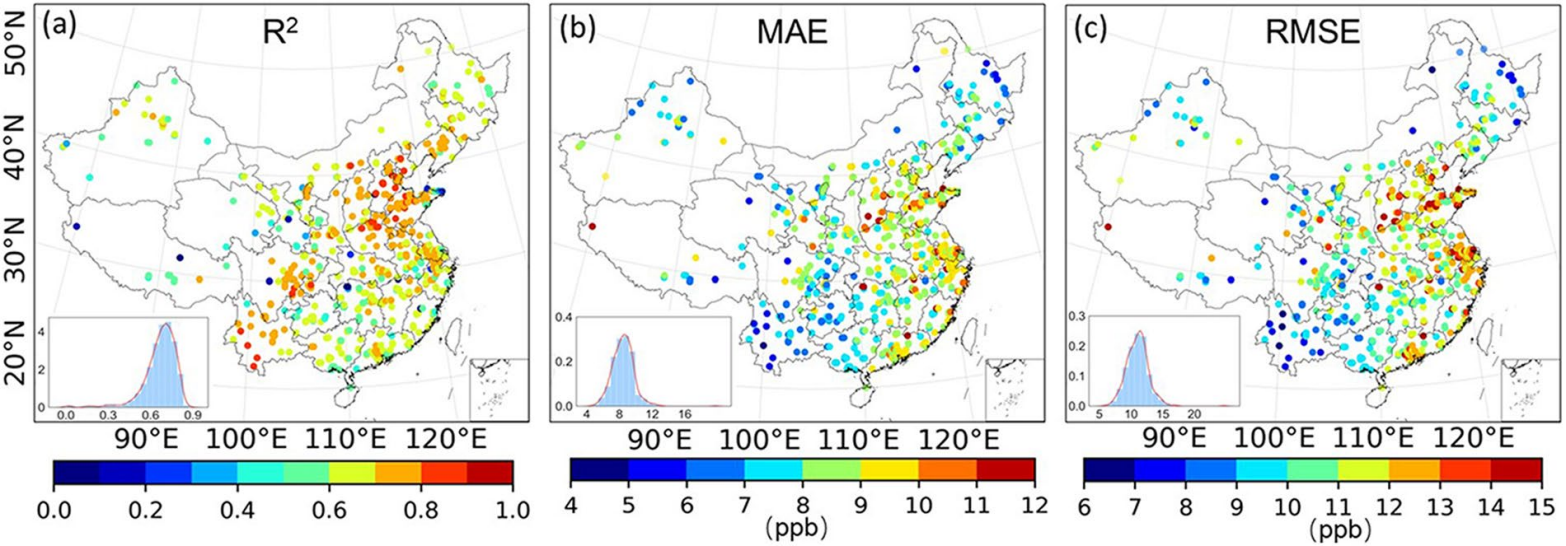
Spatial distribution and histograms with density curves of (**a**), MAE (**b**) and RMSE (**c**) of model estimated surface ozone concentrations (ppb) and observations across China in 2020.

The spatial prediction accuracy of the LSTM model was evaluated based on the values of CV R^2^ (Fig. 5a), MAE (Fig. 5b), and RMSE (Fig. 5c), which were 0.66, 8.45 ppb, and 11.03 ppb, respectively. The R^2^ values at around 75% of the monitoring sites ranged from 0.61 to 0.87, and 75% of the monitoring sites had MAE values less than 9.25 ppb and RMSE values less than 12.00 ppb. The model showed a better hourly ozone prediction ability in the North China Plain and the Southwest region of China, with R^2^ values generally higher than 0.70 (Fig. 5a). Furthermore, the MAE and RMSE values in the southwest region are lower than those in other regions (Fig. 5b,c). However, the model’s uncertainty was higher in the central and eastern regions of China, with MAE values ranging from 8.00 to 11.00 ppb and RMSE values ranging from 11.00 to 14.00 ppb.

The independent test set mainly comprised hourly ozone concentration records obtained in 2014 (June-December; 946 sites) and 2021 (January-February; 1720 sites). Supplementary Figure S8 shows the HrSOD performance (R^2^ = 0.64, RMSE = 15.44 ppb, MAE = 10.66 ppb) across China at the hourly time-scale. Despite the differences in data distribution and sample size between the test set and the validation set, the model continued to perform well on the test, suggesting that the LSTM model could accurately capture the spatiotemporal patterns of surface ozone concentrations.

**Fig. 6:**
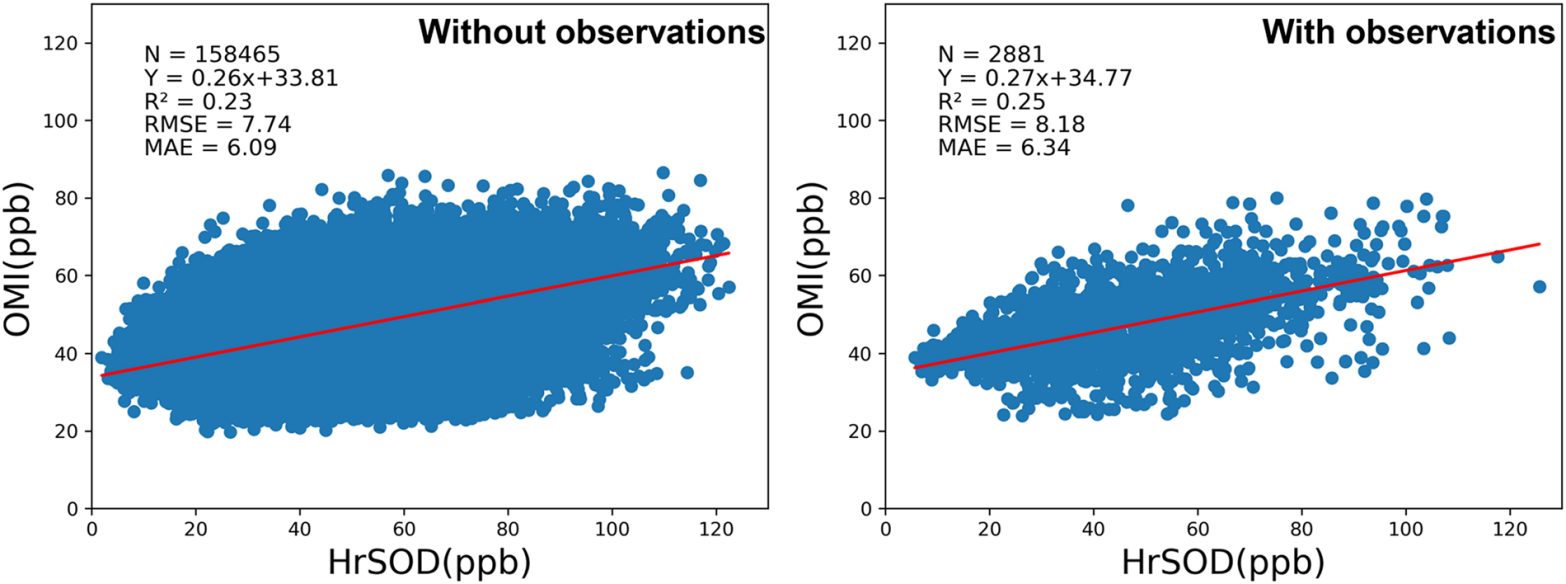
Comparisons of HrSOD against the OMI remotely sensed surface ozone concentrations across China in regions with and without measurement stations. The red lines represent linear regression lines.

In light of lacking direct observations in some regions, we figured out a workaround to validate the reliability of LSTM model. Specifically, the OMI remotely sensed surface ozone concentrations were taken as a benchmark for the whole country. Despite criticism for its low accuracy^18^, the OMI surface ozone concentration product has a consistent performance both spatially and temporally in areas with and without monitoring stations. Therefore, the OMI surface ozone concentration product is an appropriate choice for evaluating the consistency of the HrSOD product. Figure 6 shows that the HrSOD product has a highly congruous performance against the OMI product in regions with (R² = 0.25, RMSE = 8.18 ppb; nationwide) and without site measurements (R² = 0.23, RMSE = 7.74 ppb; nationwide). Hence, we can conclude that the HrSOD product demonstrates consistent performance across China. We also compared the spatial ozone patterns from HrSOD and OMI data in 2015 (Supplementary Figure S9). The result shows that except in northeastern China, HrSOD and OMI generally show a consistent pattern with higher ozone concentration in the south and lower ozone in the north. Shen *et al*.^18^ also observed this pattern in their comparison of surface ozone observations with OMI enhancements, and found that OMI data exhibits relatively weak retrieval sensitivity in the north due to greater upper tropospheric ozone variability there than in the south.

### Spatiotemporal variations of surface ozone across China

**Fig. 7:**
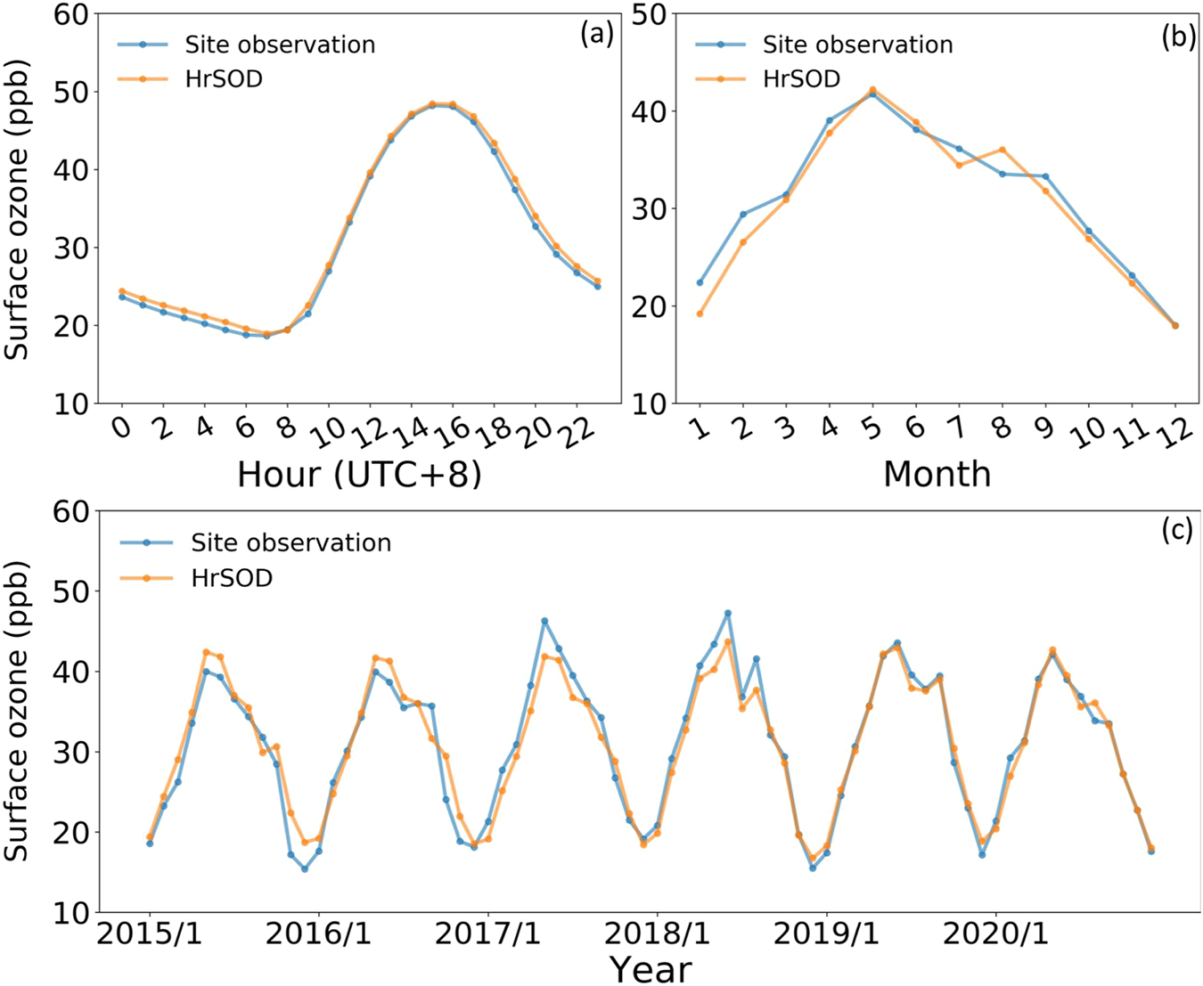
Diurnal (**a**), mean monthly (**b**) and monthly (**c**) observed surface ozone concentrations and the corresponding HrSOD values in China during 2015–2020.

The diurnal, monthly mean and monthly surface ozone concentrations predicted by the LSTM model were consistent with those observed across China from 2015 to 2020 (Fig. 7). The diurnal variations of mean hourly surface ozone concentrations across China exhibited a unimodal curve. Specifically, the national average for hourly ozone concentrations gradually increased from around 9:00–10:00 (UTC + 8) and peaked at approximately 15:00 (UTC + 8) with a value of about 48.42 ppb based on either site measurements or the HrSOD product (Fig. 7a). Then the mean hourly ozone concentrations gradually decreased to about 20.00–25.00 ppb. Similarly, the mean monthly ozone concentration in China also displayed a unimodal pattern from 2015 to 2020 (Fig. 7b). The ozone concentration gradually increased and peaked in May at 41.73 ppb. Subsequently, the concentration gradually decreased until December, when the surface ozone concentration reached its minimum at about 17.28 ppb. The surface ozone concentrations (Fig. 7c) across China showed regular seasonal changes from 2015 to 2020, and the concentrations gradually increased over this period. It is noteworthy that the ozone concentrations in May 2017 (46.30 ppb) and June 2018 (47.28 ppb) were higher than those in other months.

**Fig. 8:**
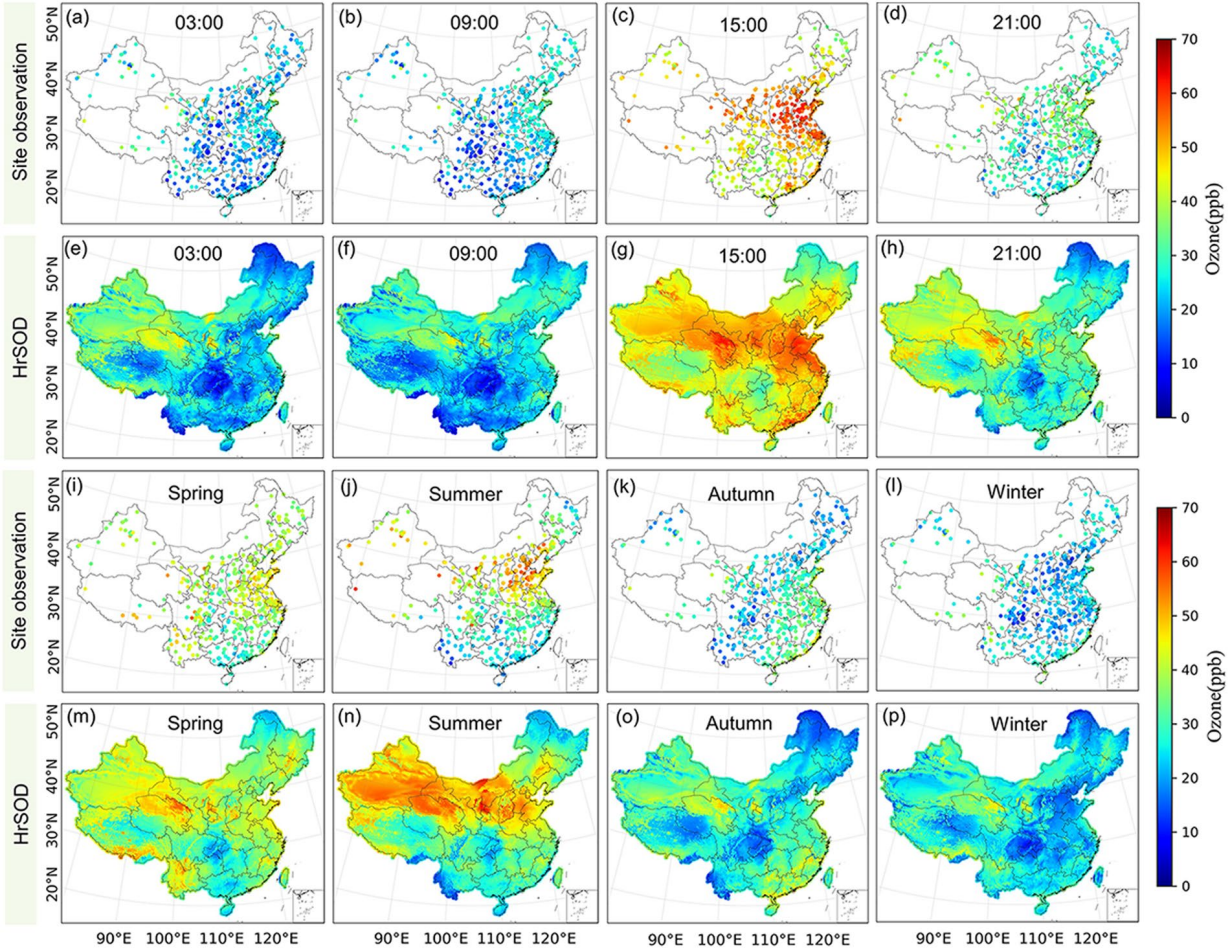
Mean hourly surface ozone concentrations at 3:00 (**a,e**), 9:00 (**b,f**), 15:00 (**c,g**) and 21:00 (**d,h**) (UTC + 8), and seasonal average surface ozone concentrations in spring (**i,m**), summer (**j,n**), autumn (**k,o**), and winter (**l,p**) from ozone observation sites and HrSOD during 2015 to 2020 across China.

Figure 8 shows that the spatial distribution of surface ozone concentrations observed cross China from 2015 to 2020 is generally consistent with HrSOD at different time scales. The highest ozone concentration was observed at 15:00 (Fig. 8c,g), while the southwestern region had lower ozone concentrations compared to the North China Plain region at all four times. The multi-year mean seasonal ozone concentrations were predicted to be 37.64 ± 3.35, 39.16 ± 2.37, 28.40 ± 3.17, and 25.07 ± 2.60 ppb in spring (March–May), summer (June–August), autumn (September–November), and winter (December, January, and February), respectively. In springs (Fig. 8i,m), northern and eastern China had higher ozone concentrations. In summer (Fig. 8j,n), the areas with high ozone concentrations (>45.00 ppb) were north China, northwestern China, and southern Inner Mongolia. The hotspot areas with high ozone concentrations in autumns (Fig. 8k,o) decreased and spread to the southeast coast. During winters (Fig. 8l,p), the areas with high ozone concentrations (>30.00 ppb) almost disappeared in southeastern China. The strong spatial and temporal variations in surface O_3_ concentrations could be attributed to multiple drivers. In densely populated regions, particularly in northern China, industrial air pollutions contribute more to O_3_ production than in other regions^79^. In contrast, in northwest China, despite relatively low population, the special topography and strong radiation affect atmospheric diffusion conditions and strengthen photochemical reactions, resulting in generally higher background surface O_3_ concentrations. In summer time, high temperature and dry air accelerates photochemical reactions in northern China^80^. Correspondingly, in southern China, due to the influences of monsoon climate in summer, frequent cloud cover and relatively high-water vapor benefit removal of surface ozone. However, surface O_3_ concentrations become higher in southern China in autumns because of intensified solar radiation during the season. In springs, stratospheric ozone intrusions and photochemical reactions of winter-accumulated precursor compounds contribute to high surface O_3_ concentrations in some regions, notably in southwest and northeast China^81^.

**Fig. 9:**
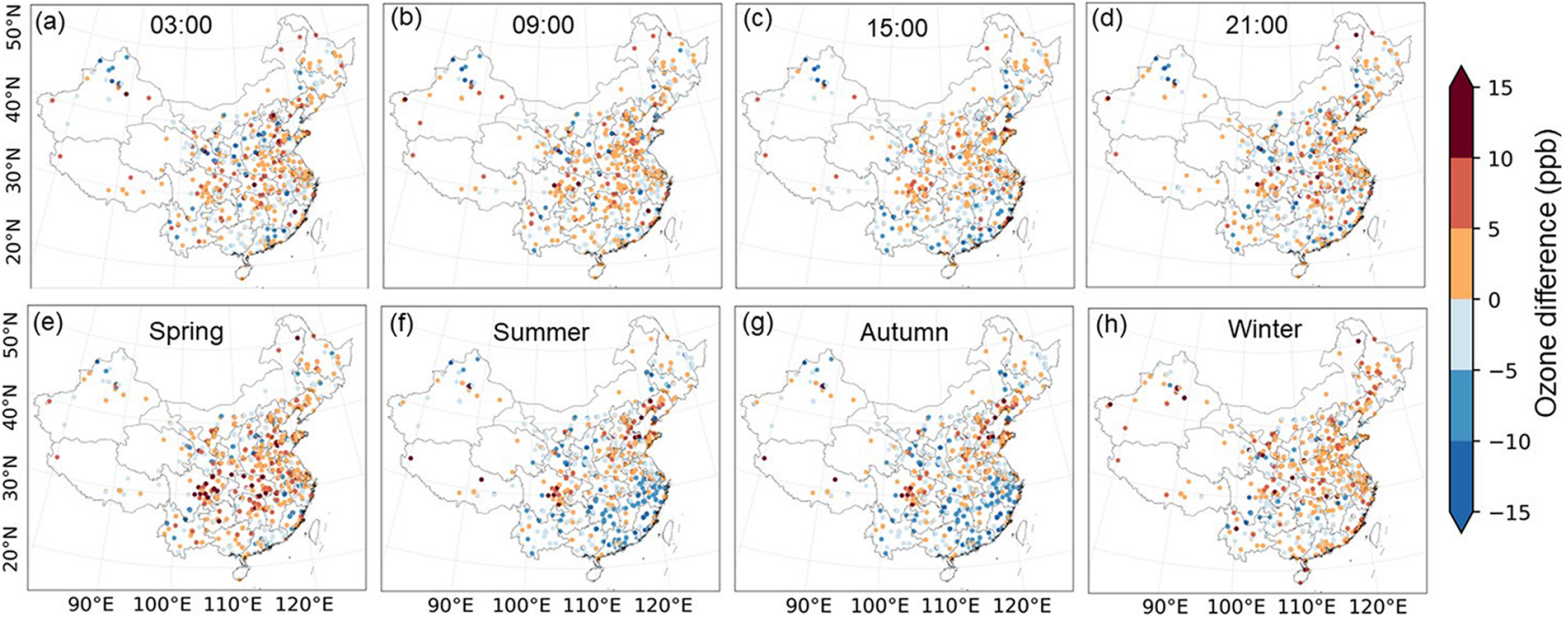
Biases in estimated mean hourly surface ozone concentrations (observed minus predicted) at 3:00 (**a**), 9:00 (**b**), 15:00 (**c**), and 21:00 (**d**) (UTC + 8) and seasonal mean of hourly surface ozone concentrations in spring (**i,m**), summer (**j,n**), autumn (**k,o**), and winter (**l,p**) during 2015–2020 across China.

Upon comparing predicted and observed mean hourly surface ozone concentrations, The discrepancies mostly fell within a range of −5 to 5 ppb at the hourly scale. The HrSOD estimates tended to underestimate hourly surface ozone concentrations in the majority of China, except some overestimation in the southeast part. Such overestimation was particularly manifest during summers and autumns, with the bias reaching up to approximately 5–10 ppb (Fig. 9f,g).

### Surface ozone changes in key regions

**Fig. 10:**
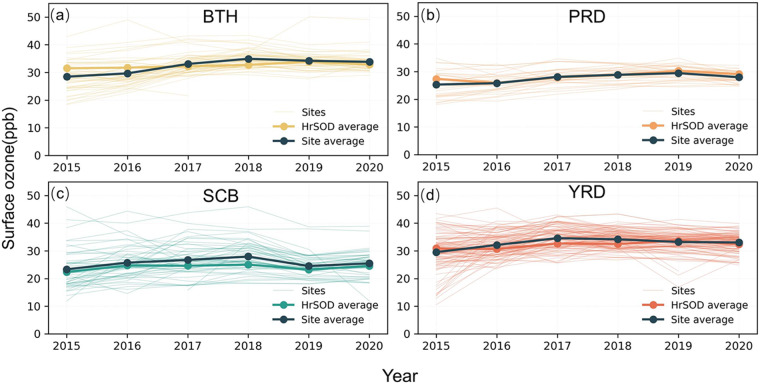
Temporal dynamics of mean annual mean surface ozone concentrations in the BTH (**a**), PRD (**b**), SCB (**c**), YRD (**d**). BTH: Beijing-Tianjin-Hebei region; SCB: Sichuan Basin; PRD: Pearl River Delta; YRD: Yangtze River Delta.

Among the four megacity clusters, mean annual surface ozone concentrations in BTH (mean = 32.35 ppb), YRD (mean = 32.78 ppb), and PRD (mean = 27.59 ppb) regions were higher than in the SCB (mean = 25.62 ppb) region during 2015–2020. In the BTH region (Fig. 10a), surface ozone concentrations showed a continuous increase from 28.45 ppb in 2015 to 34.92 ppb in 2018, before decreasing to 33.80 ppb in 2020. In the PRD and YRD regions (Fig. 10c,d), the annual mean surface ozone concentrations showed an obvious increase from 25.32 and 29.54 ppb in 2015 to 34.58 ppb in 2017 and 29.45 ppb in 2019, respectively, and then declined to 33.03 ppb and 28.00 ppb in 2020. Similar to BTH, both regions experienced a growth in surface ozone concentrations before 2017. In contrast, annual mean surface ozone concentrations in the SCB region were relatively low, which showed an increase from 23.35 ppb in 2015 to 27.95 ppb in 2018, a decrease in 2019, followed by a slight rebound in 2020.

The seasonal patterns of surface ozone concentrations varied across the four key regions (Supplementary Figure S10a). From April to July, the monthly mean ozone concentrations were higher than 38.00 ppb across China, while they were less than 24.00 ppb in January, November, and December. In BTH, the ozone concentrations followed a unimodal distribution and gradually increased over time, peaking in June (57.30 ppb) before declining to their lowest value in December (11.81 ppb). Conversely, the other three regions (YRD, SCB, and PRD) showed a bimodal pattern, with the first peak occurring in May (44.22 ppb in YRD, 29.64 ppb in PRD, and 37.80 ppb in SCB), and the second peak occurring in September (40.09 ppb in YRD), October (36.02 ppb in PRD), and August (38.95 ppb in SCB), respectively. The lowest surface ozone concentrations were found to be 16.31 ppb (YRD in December), 22.25 ppb (PRD in January), and 11.45 ppb (SCB in December).

We conducted additional partial correlation analysis to investigate the relationships between regional surface ozone concentrations and meteorological factors at hour scales (Supplementary Figure S10b). The results indicate that temperature and relative humidity are the primary controlling factors of regional surface ozone concentration at the hourly scale. Besides BTH, radiation is relatively important. Relative humidity dominates in the YRD region, horizontal wind speed, temperature, and relative humidity co-regulated surface ozone concentration in the PRD region, and solar radiation dominated in the BTH region.

### Comparison with previous studies

We conducted a comparison between HrSOD and two other datasets: the long-term hourly surface ozone mixing ratios dataset (1.25° × 1.875°) estimated by the UK Earth System Model 1-0-LL (UKESM1-0-LL) under the Coupled Model Intercomparison Project Phase 6 (CMIP6), and the ERA5 reanalysis hourly surface ozone dataset simulated by the atmospheric model at a resolution of 0.25° × 0.25°. The validation results are presented in Supplementary Figure S11. It is obvious that there exist large uncertainties in ozone estimates from ERA5 and CMIP6. The CMIP6 datasets (R^2^ = 0.01, RMSE = 32.06 ppb and MAE = 25.77 ppb) exhibited an overestimation of surface ozone concentrations in western China and underestimated ozone concentrations in the North China Plain (Supplementary Figure S12a), and mainly due to uncertainty in emission inventories, deposition processes or vertical mixing^82^. Similarly, the ERA5 simulated surface ozone dataset (R^2^ = 0.01, RMSE = 54.19 ppb and MAE = 49.97 ppb) showed significant deviations from the observed values and displayed a decreasing trend from north to south (Supplementary Figure S12b). This discrepancy primarily arises from the simplified representation of ozone chemistry mechanisms in the ERA5 simulations^83^. In contrast, our HrSOD product (Supplementary Figure S12c) demonstrated a significant improvement compared to the aforementioned products, exhibiting high consistency with sites measurements (e.g., R^2^ = 0.71, RMSE = 11.14 ppb and MSE = 8.44 ppb). A notable phenomenon is the comparison of surface ozone concentration in Tibet against Qinghai or Xinjiang regions. While Wei *et al*.^20^ reported that ozone concentrations over the Tibet were comparable to those in Xinjiang and Qinghai, many studies^19,35,84^ align with this study. Even seen from the limited number of ground observations in Tibet, the central and western parts of Tibet had much lower surface ozone concentrations compared to Xinjiang and northeast Qinghai. The OMI remote sensing data can further support this phenomenon. After all, both natural and anthropogenic conditions are so different in Tibet from those in Xinjiang and northeast Qinghai. Such a difference is more likely to be caused by the different algorithms and more ground observations are needed in areas of data scarcity to constrain estimation uncertainties in surface ozone concentrations.

In traditional approaches for predicting surface ozone concentrations, meteorological variables and ozone precursors such as NO_2_ and VOCs emissions inventory data have been commonly included^19,20^. While these data have played a significant role in predicting ozone concentrations, it is worth noting that in our study, we did not utilize these specific data as predictive variables. Instead, we relied mainly on hourly and daily satellite retrievals and some auxiliary information (population and GDP data), and the results proved that this strategy of parsimonious inputs could obtain satisfying outcomes. This result may be explained by the estimation of VOCs and NO_X_ emissions inventory data, which is mainly obtained by emission inventory models using emission factors for different emission sources, including power plants, industrial plants, as well as residential, transportation, and agricultural sectors^85^. Therefore, there may be a strong correlation between ozone precursor emission data and population and GDP distribution data, which may cause data redundancy if they are used as predictor variables simultaneously.

Moreover, there exist scale effects for different environmental variables influencing surface ozone concentrations. At hourly time-scale (diurnal), meteorological conditions, such as radiation, air temperature and air humidity, are critical in ozone formation and destruction^22,32^. In contrast, precursor emissions^19^, warming^86^ and climate patterns^87^ play more important roles in regulating surface ozone concentrations at daily, seasonal and yearly time-scales. Thus, in the case of adopting instantaneous and particularly the daily remotely sensed ozone products, which have implicitly reflected the long-term changes in emission levels of ozone precursors, there is no necessity to include ozone precursor emissions in the predictive model. In addition, ozone precursor emission data are mostly monthly scale data with low spatial resolution, and due to the diversity of emission sources and the lack of reliable measurement methods, the data uncertainty is high, and it contributes relatively low in the previous ozone prediction models^19^. In future research, obtaining high-resolution and high-precision data on ozone precursors may become a key focus and direction for ozone estimation. As for whether this finding is limited to ozone or also occurs to other air pollutants, e.g., PM_2.5_, it is a topic worth discussing. At the hourly to daily time-scales, wind speed and local emissions are the major players in determining PM_2.5_ concentrations^88^. Compared to gaseous atmospheric pollutants (such as SO_2_, NO_2_, O_3_ and NH_3_, which are PM_2.5_ precursors), impacts of meteorological conditions (except wind) on PM_2.5_ are relatively minor in China^89^, which is exactly opposite to those found for ozone^20,85^. Thus, it is reasonable to conclude that influencing factors critical in predicting different air pollutants at an hourly time-scale should be different.

### The driving factors for surface ozone concentrations in key regions

Short-term changes in ozone concentration are particularly sensitive to meteorological conditions^54,90^. For example, at hourly scale, surface ozone concentrations are significantly influenced by different meteorological conditions and synoptic type, especially strong solar radiation, high temperature and high humidity, which play a key role in photochemical reactions involved in ozone formation^32,91^. Here relative humidity is identified as the most influential variable in YRD and PRD, air temperature is the most influential variable in BTH, while the two factors show comparable effects in SCB. Notably, the positive surface ozone concentration-air temperature relationship may become increasingly important in the context of global warming, which could lead to an increase in surface ozone concentrations, i.e., “ozone climate penalty”^91^. For example, Wu *et al*.^86^ estimated that ozone levels will increase by 2–5 ppb in a warmer environment by 2050. The mechanism lies in that warming can lead to increased emissions of biogenic VOCs from natural sources, accelerate peroxyacetyl nitrate dissociation, enhance natural soil NOx emissions, influence the efficiency of ozone dry deposition, and thus finally affect atmospheric ozone dynamics^87^.

In 2020 when COVID-19 lockdown/control occurred, surface ozone dynamics differed in the four major megacity regions. Specifically, surface ozone concentrations in the PRD region decreased, primarily due to a reduction in anthropogenic emissions over a short period and the influence of decreased solar radiation intensity^92^. This result is consistent with previous research, as reduction in NOx emissions from road traffic could promote surface ozone concentration (NO titration effect; NO + O_3_ = NO_2_ + O_2_). Moreover, lower emissions of inhalable particulate matter, along with higher solar radiation, favor ozone formation^93,94^. However, this mechanism cannot explain surface ozone concentration changes in all regions. In YRD and BTH regions, surface ozone concentration remained stable, while it even experienced an increase in the PRD region in 2020. This phenomenon suggests that the interactive effects of various natural and anthropogenic factors affecting surface ozone concentrations are complex and efforts for reducing surface ozone pollution should account for this.

### Uncertainties and limitations

In this research, uncertainties exist in several aspects. Firstly, the monitoring stations were mainly concentrated in the central-eastern region of China, which may limit the model’s ability to fully capture the relationship between surface ozone concentrations and environmental factors in western China. Additionally, the majority of monitoring stations were located in urban areas, which may restrict the model’s accuracy in estimating surface ozone concentrations in natural and agricultural ecosystems. A typical example is the Taklimakan Desert in northwestern China, which is enclosed by high mountains. Due to the accumulation of ozone precursors emitted from surrounding oasis areas^95^, surface ozone concentration in this region was relatively high (Supplementary Figure S9) but with its temporal variations more related to natural factors, including solar radiation^96^ and air temperature^97^. In comparison with site observations from the center of the desert (Tazhong station, 38°58′N, 83°39′E), the HrSOD values (Fig. 8f,g) were consistent with the observed maximum and minimum hourly surface ozone concentrations (69.2 ppb and less than 20 ppb, respectively, during July 2010-Dec 2017^97^), and the mean daily surface ozone concentration (49.0 ppb during June 2010-March 2012^96^ *vs*. 51.5 ppb by HrSOD). Nevertheless, the HrSOD could not fully capture the temporal variations at the station during 2010–2017. For instance, in 2015, the mean annual surface ozone concentration estimated by HrSOD (54.6 ppb; Supplementary Figure S9) was rather higher than the observations^97^. Thus, more extensive and continuing observations are required in the future to improve the accuracy of surface ozone predictions, particularly in regions of data scarcity. Secondly, uncertainties can arise from the input data. For example, ERA5 reanalysis data underestimates surface temperatures in the coastal urban agglomerations of southeast China and the Tibetan Plateau^66,98^, which may lead the model to underestimate ozone concentrations. Enhancing the accuracy of meteorological data, land use maps, and socio-economic data is necessary to further improve ozone estimation accuracy. Furthermore, the mismatch in temporal resolution between OMI remote sensing data and ozone measurements may also affect the final estimation accuracy.

While the LSTM networks effectively capture the temporal variations of surface ozone concentrations, spatial information such as changes in pollutant concentrations due to the emission and transport of surrounding pollutants is not fully considered. The underestimation of the LSTM model in southeast China but overestimation in other parts (Figs. 8, 9) also underlines there may exist some deficit in the trained model in capturing the spatial heterogeneity of surface ozone concentrations across different environmental conditions. Therefore, to enhance the current deep learning model, combining it with other algorithms that could effectively extract spatial dependencies within data may be beneficial. The most widely used framework is integrating CNN with LSTM to leverage the strengths of temporal memory by LSTM and feature representation by CNN for improved prediction accuracy. To validate the plausibility of this methodology, we conducted extra simulations using the Convolutional Long Short-Term Memory (ConvLSTM) algorithm. However, ConvLSTM performed only slightly better than LSTM, at the cost of much more model parameters and computation resource consumption (Supplementary Table S3) and with the spatial biases not resolved (Supplementary Figure S13). This unexpected result suggests that more efforts are warranted in developing novel algorithms to address the fundamental challenge in considering both spatial and temporal information inherently embedded in environmental datasets.

### Potential applications of HrSOD

Compared to the currently available surface ozone products in China, HrSOD offers several advantages. It covers a longer time range and has a higher temporal resolution, enabling more robust historical environmental impact and human health risk assessments. HrSOD can be used to derive various ozone exposure indicators (Supplementary Figure S14), such as seasonal 7-hour mean ozone concentrations (M7), seasonal 12-hour mean ozone concentrations^99^ (M12), sum of all hourly average concentrations >60 μg kg^−1^ (SUM06)^100^, cumulative ozone exposure index based on sigmoid-weighted daytime ozone concentrations^101^ (W126), and accumulated hourly ozone concentration over a threshold of X μg kg^−1^ during daylight hours^102^ (AOTX). Therefore, HrSOD can cater to the requirements of ozone impact models and provide flexibility for assessing ozone effects on ecosystems^38^ and epidemiological studies^103^.

### Supplementary information

Supplementary information

## Data Availability

The code is available on GitHub (https://github.com/Wenxiu0902/Ozone_prediction) primarily using Python and R languages. It includes data preprocessing, model training, testing, prediction, and visualization sections. Additionally, sample model input data is also provided.
