# Supplementary material for: Parsimonious estimation of hourly surface ozone concentration across China during 2015–2020
Source: Sci Data. 2024 May 14;11:492. doi: 10.1038/s41597-024-03302-3 (PMC11094007; doi:10.1038/s41597-024-03302-3)
Supplement: Supplementary file 1 — Supplementary information [file 41597_2024_3302_MOESM1_ESM.pdf]

Content

Table S1 2
Table S2 3
Table S3 3
Figure S1 4
Figure S2 4
Figure S3 4
Figure S4 5
Figure S5 5
Figure S6 6
Figure S7 6
Figure S8 7
Figure S9 7
Figure S10 7
Figure S11 8
Figure S12 8
Figure S13 8
Figure S14 9

Table S1. Summary of the data sources used in this study.

Category	Variable	Unit	Spatial resolution	Temporal resolution	Source
Ground measurements	O_3: Ozone	μg m^{-3} convert to ppb	–	Hourly	CNEMC
Climate data	DSR: Downward shortwave radiation<br>RH: Relative humidity<br>TEM: 2-m air temperature<br>SP: Surface pressure<br>WU: 10m u-component of wind<br>WV: 10m v-component of wind<br>PRE: Precipitation	W m^{-2}<br>%<br>K<br>hPa<br>m s^{-1}<br>m s^{-1}<br>mm	0.1° × 0.1°	Hourly	ERA5-Land reanalysis
Socio-economic data	POP: Population	One person per grid	1 km × 1 km	5 years	Resource and Environment Science and Data Center
	GDP: Gross Domestic Product	10,000 yuan per grid			
Satellite remote sensing data	TO_3: Total column ozone	DU	0.25° × 0.25°	Daily	OMI/Aura products
	SFO_3: Surface ozone concentrations	DU	13 km × 24 km	Instantaneous	
	Land use: Forest \ Grassland \ Urban land \ Cropland		0.05°× 0.05°	Annual	MODIS products
Other	LAT: latitude	Degree	0.1° × 0.1°		
	LON: longitude	Degree	0.1° × 0.1°		

Table S2. Detailed results of model tests at eight lookback windows.

Lookback windows (hour)	R^2	RMSE (ppb)
1	0.69	14.49
2	0.68	14.74
6	0.73	13.56
12	0.74	13.22
**24**	**0.75**	**12.84**
48	0.74	13.09
72	0.74	13.13

Table S3. Comparison between different models. The year 2015 was selected with an 80% training and 20% testing data split.

Metric	ConvLSTM	LSTM
Parameters (K)	1195.21	13.60
GFLOPs	32.09	0.029
RMSE	9.88	10.58
R^2	0.70	0.68
MAE	7.48	8.14

Parameters refers to the total number of trainable parameters in a neural network model, used to measure the model's complexity and capacity. **GFLOPs** refers to the total number of floating-point operations performed by the model, used to measure the model's computational requirements and complexity (1 GFLOPs=10^9 FLOPs).

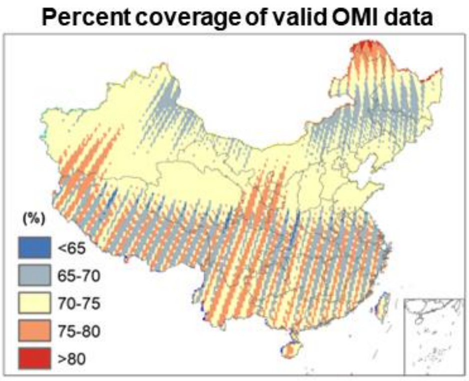

Figure S1. Mean annual percentage of days with valid OMI data per grid cell from 2015 to 2020.

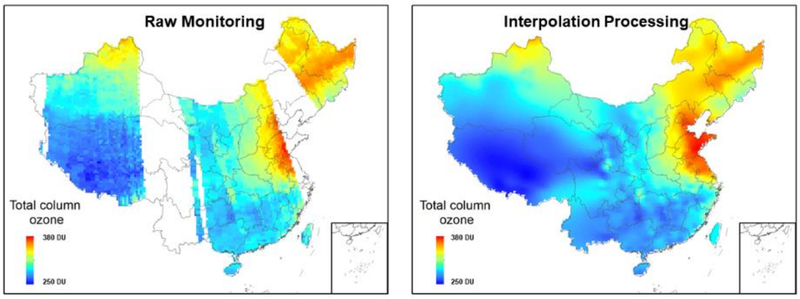

Figure S2. Comparison between original and filled OMI ozone column concentrations on July 1, 2018.

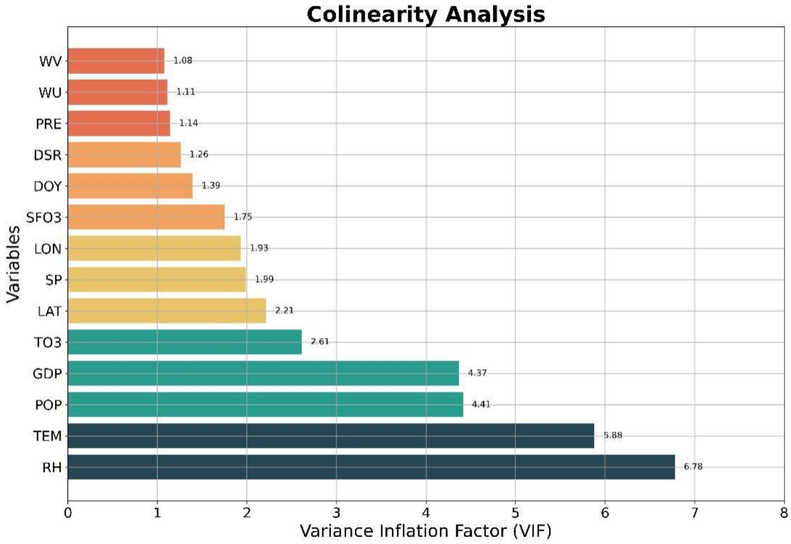

Figure S3. Collinearity analysis for the input variables used in the LSTM model. Predictor variables with a variance inflation factor (VIF) value less than 8 were retained. (WV: 10-m v-component of wind; WU: 10-m u-component of wind; PRE: Precipitation; DSR: Downwelling surface radiation; DOY: Day of year; SFO_3: Surface ozone concentration; Lon: longitude; SP: Surface pressure; Lat: Latitude; TO_3: Total column ozone; GDP: Gross domestic product; POP: Population; TEM: 2-m air temperature; RH: Relative humidity).

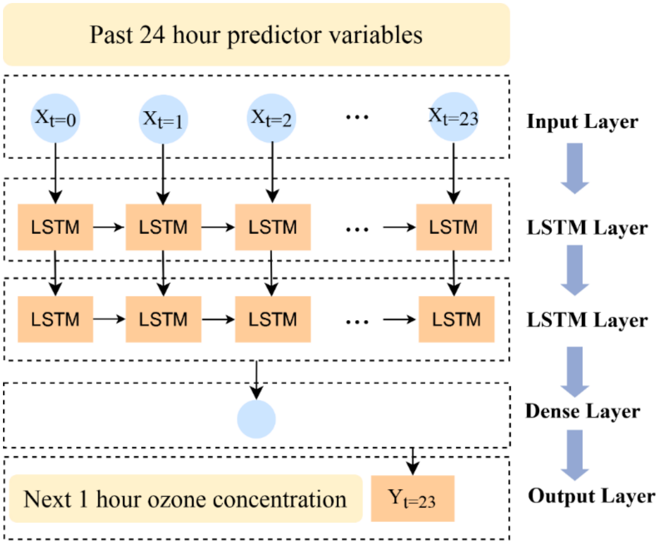

Figure S4. Long Short-Term Memory (LSTM) model structure.

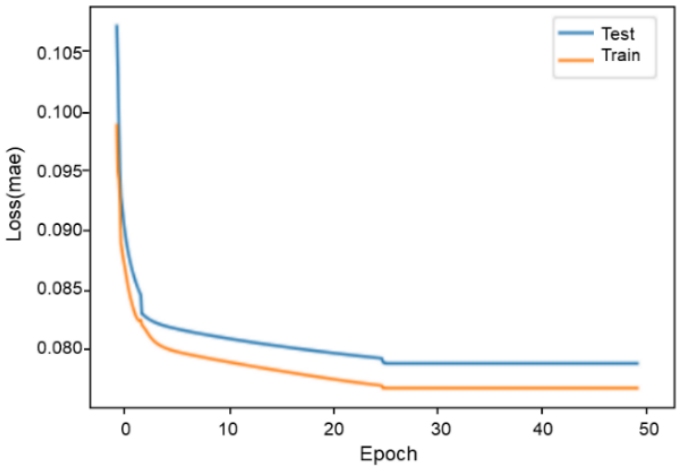

Figure S5. Loss functions of the LSTM model for training data and testing data, respectively, at each epoch.

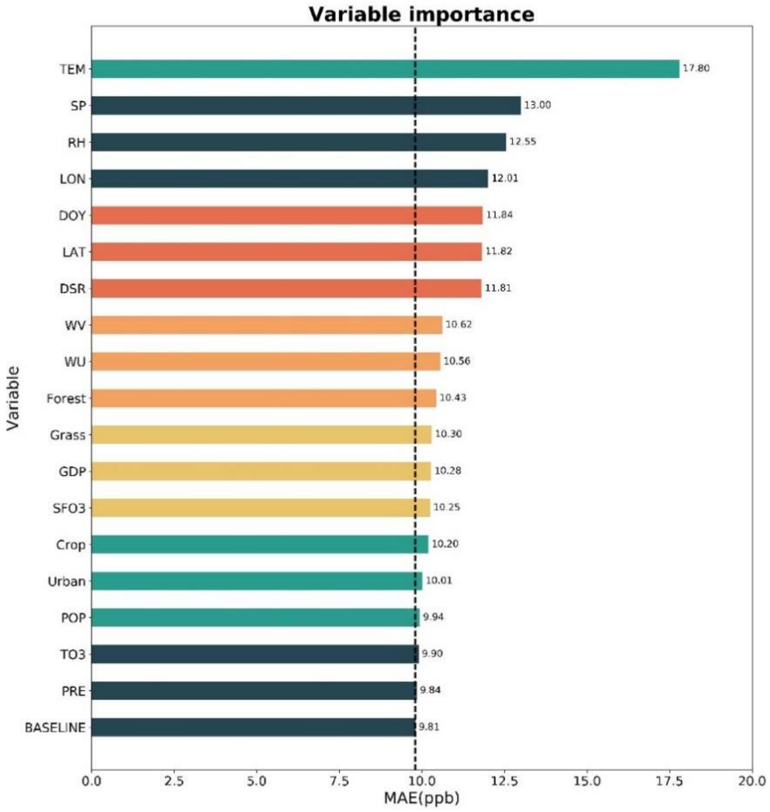

Figure S6. The variable importance values of key variables for ozone estimates. (TEM: 2-m air temperature; SP: Surface pressure; RH: Relative humidity; LON: Longitude; DOY: Day of year; LAT: Latitude; DSR: Downwelling surface radiation; WV: 10-m v-component of wind; WU: 10-m u-component of wind; POP: Population; GDP: Gross domestic production; SFO_3: Surface ozone concentration; TO_3: Total column ozone; PRE: Precipitation)

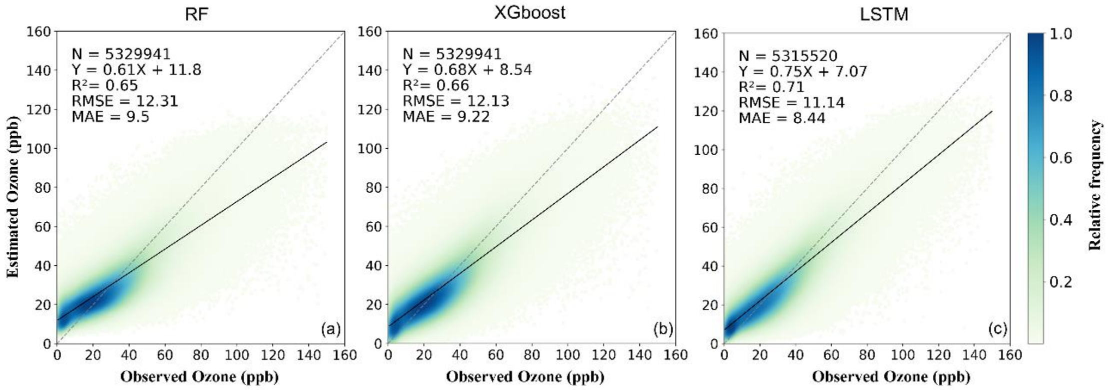

Figure S7. Comparison of the LSTM algorithm (c) with Random Forest (a) and XGboost algorithms (b). The training data is from 2015 to 2019, and the test data is from 2020. All the three algorithms use the same input variables as described in the manuscript.

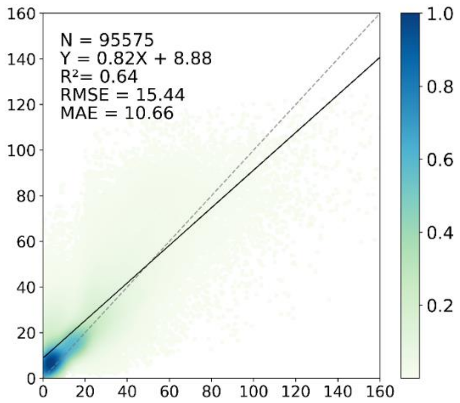

Figure S8. Comparisons between model estimated surface ozone concentrations and observations in years 2014 and 2021 across China.

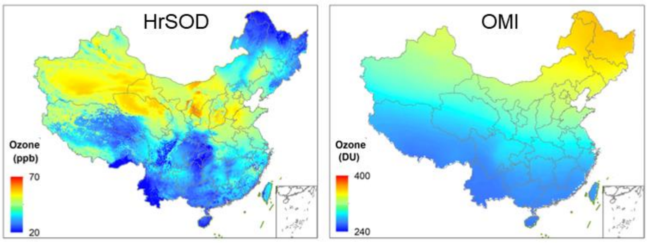

Figure S9. Spatial patterns of ozone concentrations from HrSOD and OMI remotely sensed products across China in 2015.

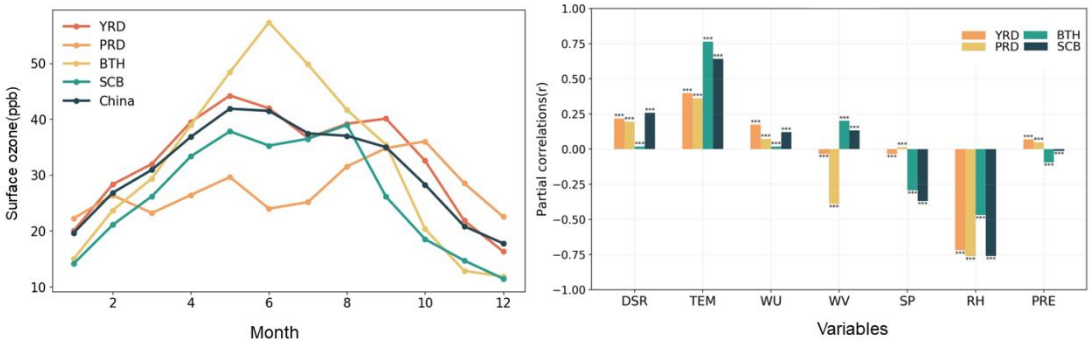

*** $p < 0.001$ level.

Figure S10. Mean monthly surface ozone concentrations and partial correlations between regional surface ozone concentrations and meteorological factors at hour scales in four megacity clusters in the BTH, PRD, SCB, YRD. BTH: Beijing-Tianjin-Hebei region; SCB: Sichuan Basin; PRD: Pearl River Delta; YRD: Yangtze River Delta.

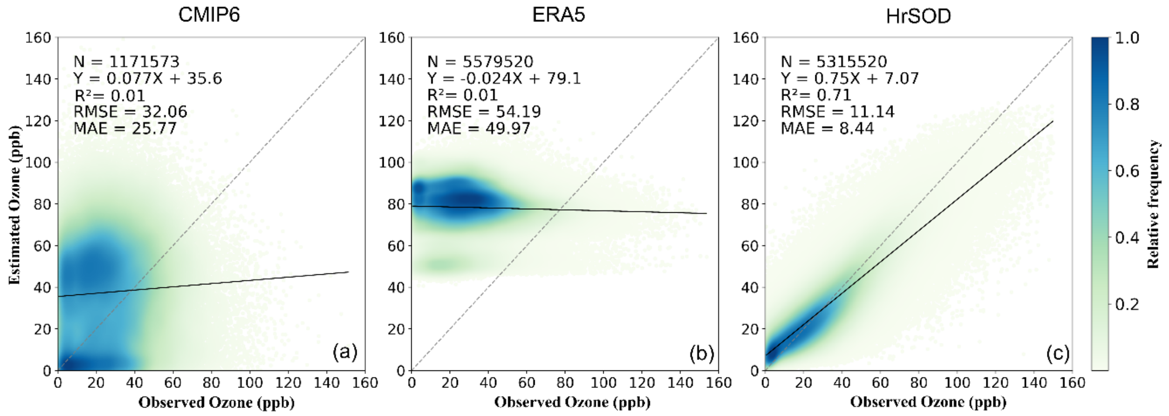

Figure S11. Comparisons between estimated surface ozone concentrations from CMIP6 (a), ERA5 (b), and HrSOD (c) products and observations across China in 2020.

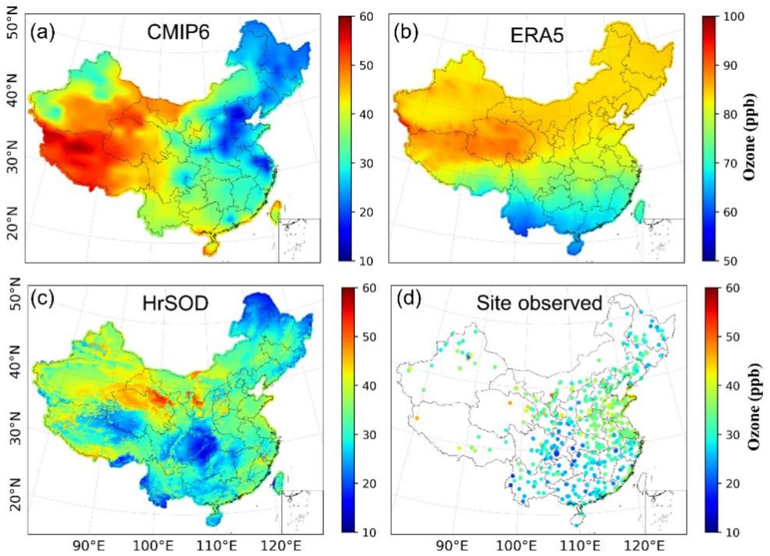

Figure S12. Mean annual O_3 concentrations from CMIP6 (a), ERA5 (b), and HrSOD (c) products and ozone observation sites (d) across China in 2020.

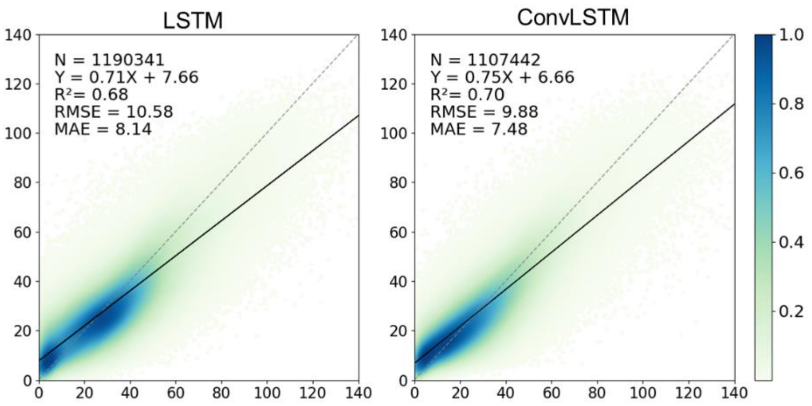

Figure S13. Comparison of the LSTM algorithm with ConvLSTM algorithms. The year 2015 was selected with an 80% training and 20% testing data split.

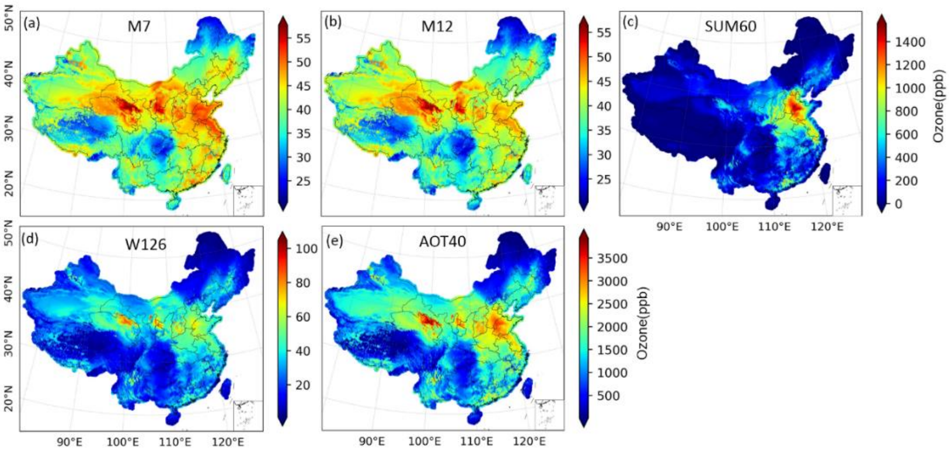

Figure S14. Spatial distribution of different ozone exposure indicators calculated by HrSOD across China in 2020, including M7 (a), M12 (b), SUM06 (c), W126 (d) and AOT40(e).
